# Survey on the Working Conditions, Salary, and Job Satisfaction of Employed Veterinarians in Germany

**DOI:** 10.3390/vetsci13050494

**Published:** 2026-05-19

**Authors:** Katharina Charlotte Jensen, Christian Wunderlich, Lilith Steingräber, Martina Warschau, Maren Ewert, Elisabeth Brandebusemeyer

**Affiliations:** 1 Association of Employed Veterinarians, 30612 Hannover, Germany; 2 Institute of Veterinary Epidemiology and Biostatistics, School of Veterinary Medicine, Freie Universität Berlin, 14163 Berlin, Germany

**Keywords:** veterinarians, working conditions, salary, wage, work protection, job satisfaction, corporatization, gender pay gap

## Abstract

This online survey aimed to elaborate on the salary, working conditions, and job satisfaction of employed veterinarians in Germany. The focus was on factors influencing salaries, violations of German laws, and comparisons between employees of owner- and corporate-managed practices. Answers of up to 1184 veterinarians were analyzed, representing 6% of employed veterinarians. The hourly salary increased by around 19% compared to a study in 2020, but remained significantly lower than in comparable professions and did not rise as much as the national average over the last five years. A multifactorial linear model showed that working experience, additional qualifications, leadership role for other veterinarians, section (pets, equines, farm animals, or non-curative), and gender significantly influenced the salary. The adjusted gender pay gap was about 7%. Employees of corporations earned significantly more than veterinarians being employed in owner-managed practices, but not when salary was adjusted for overtime. Moreover, employees of corporations had significantly lower job satisfaction. Requirements of the German Working Hours Act were regularly not complied with, as e.g., around 40% of respondents reported not being able to take their legally required break at least once per week. Results indicate that, despite improvements, there is still a need to address working conditions to retain veterinarians in the profession.

## Simple Summary

Employed veterinarians in Germany were surveyed regarding their income, job satisfaction, and working conditions via an open online survey. Answers of up to 1184 veterinarians were analyzed, representing 6% of employed veterinarians in Germany. The hourly salary for veterinarians in their first year of employment was about 21.35 € before taxes. This salary is significantly lower than the income of human medical doctors in their first year of specialization training in Germany. Section of work (pets, equines, farm animals, or non-curative), years in the profession, leadership role, and additional qualifications influenced the salary. Adjusted for these factors, men still earned around 7% more than women. Job satisfaction was comparable to that of the validation study population but lower than in other academic professions. Employees of corporates had a significantly lower job satisfaction than employees of owner-managed practices. Results indicate that requirements of the German Working Hours Act are regularly not complied with, yet around 50% of affected veterinarians were not bothered by working long hours or without breaks. Collective agreements would legally allow for longer working hours while compensating for this effort and reducing the gender pay gap.

## 1. Introduction

The working conditions and salaries of employed veterinarians in Germany, as in other countries, are often significantly worse than those of other academic professions [1,2,3]. This is associated with lower job satisfaction [4]. On the one hand, these factors lead to an exodus from the profession [2,5], which can cause or exacerbate a shortage of veterinarians. At the same time, various studies have repeatedly shown that working conditions are risk factors for increased levels of stress, depression, and suicidal ideation [6,7].

Five years ago, the Association of Employed Veterinarians (Bund angestellter Tierärzte e.V.; BaT), an association that represents the interests of employed veterinarians in Germany, and the Association of Owner-managed Small Animal Clinics (Verbund unabhängiger Kleintierkliniken; VUK) surveyed employed veterinarians in Germany [8,9]. The study showed that the gross salary of employed veterinarians (EV), at €20.51 per hour, was significantly below that of comparable professions [9,10]. There was considerable variation in job satisfaction [8]. Associations with working conditions were found: curative work, more emergency services, fewer vacation days, and lower salaries were associated with lower job satisfaction [8]. Finally, there was a significant gender pay gap and evidence that the provisions of the Working Hours Act (WHA) were regularly not complied with [9]. The WHA specifies how long an employee may work per day (generally eight hours, maximum ten hours), that a break of at least 30 min must be taken for working hours exceeding six hours, and that a rest period of at least eleven hours must be ensured after a working day [11]. Finally, it also stipulates that employers must ensure that working hours are documented [11]. Efforts to relax the WHA for veterinary medicine have not been successful so far. The widespread establishment of collective agreements that enable exemptions from the WHA has also not yet been implemented in this profession in Germany.

Since the last survey, there have been changes in society both as a whole and in veterinary medicine. The number of pets kept in Germany has remained stable since 2018, at around 34 million [12], while the number of farm animals has fallen slightly [13]. In Germany, unlike in the UK, emergency services are usually not provided by special teams, but by the veterinarians who also work at the facility during the day. Clinics are required to offer emergency services around the clock, while practices usually rotate in cooperation with other practices. As the number of veterinary clinics has continued to decline and there is no longer a single clinic in some federal states [14,15], an emergency service obligation has been introduced in all federal states [16]. This measure became necessary to ensure medical care for animals outside regular office hours.

In Germany, the costs of veterinary treatment are regulated by the Fee Schedule for Veterinarians (Gebührenordnung für Tierärzte, GOT [17]). The GOT specifies a minimum and maximum amount for all examinations and treatments. Prices above or below this fee are only permissible in justified exceptional cases. Depending on the effort involved and the time of day, veterinarians can charge a rate from factor 1 up to factor 3, with 0.1 steps in between. After the last fundamental revision took place in 1999, a new GOT was adopted in November 2022 [17]. This amendment was preceded by a study in which individual veterinary services were evaluated and measures for structural revision were proposed [18]. However, this study was conducted in 2020. Between the completion of the study and the entry into force of the amendment to the GOT, the consumer price index rose by 10% [19]. The price adjustment, therefore, affected animal owners at a time when they had also experienced significant price increases in other areas of life. At the same time, the calculated prices for individual veterinary services were already outdated when they were introduced. Operating and living costs had also risen for veterinarians, making price adjustments and salary increases or higher private withdrawals all the more important.

The third development in German veterinary medicine during this period was the continued increase in corporatization: national and international companies (hereinafter referred to as corporates or corporations) purchased additional veterinary facilities and increased their market presence, particularly in the small animal and equine sectors [20]. This development is already more advanced in other European countries [21]. Nevertheless, it remains unclear whether working for a corporation results in better working conditions or higher job satisfaction for employed veterinarians, as recent studies have shown diverging results. A recent study by the Federation of Veterinarian of Europe (FVE) found no differences in working hours at the European level, but veterinarians working for a corporate had more vacation days [20]. In another study [22], employees of corporates received more benefits but reported a lower job satisfaction. Further insights are needed here to retrieve recommendations for corporate- and owner-managed practices.

Against this background, the aim of this study was to provide an update on the situation of employed veterinarians in Germany. Based on the 2020 survey, a new survey was conducted among employed veterinarians to assess current working conditions, salaries, and job satisfaction. This study focuses, in particular, on comparing the different areas of the profession and on comparing employees of corporate- and owner-managed practices. It also examines compliance with the WHA.

## 2. Materials and Methods

### 2.1. Survey

The ethical soundness of this study was certified by the Central Ethics Committee of Freie Universität Berlin (No.: ZEA25047). Before participants could answer the first question, they were informed about the study’s objectives and had to actively agree to the privacy policy.

The questionnaire was developed based on the previous study, implemented in LimeSurvey Cloud© (Hamburg, Germany), and tested by the authors and other veterinarians from the association. Subsequently, only minor changes were made. The final version was activated on 19 September 2025, and could be answered until 2 December 2025. The invitation to participate was distributed via the following channels:

- Social media (WhatsApp status, Facebook, Instagram)
- BaT email-newsletter
- Email newsletters from the state veterinary associations of Brandenburg, Westphalia-Lippe, Saxony, Schleswig-Holstein, and Lower Saxony
- Advertisement in the German Veterinary Journal, a print journal sent to every veterinarian in Germany monthly
- Emails to professional organizations, including corporations
- Events and conferences.

### 2.2. Questionnaire

The survey consisted of 50 questions, and the questionnaire is included as Supplementary Material File S1 (original language) and in English (File S2). Fifteen of the questions were conditional questions that could only be answered if a specific answer had been given previously. This meant that the maximum number of questions a person could answer was less than 50. It took an average of 8 min (median) to complete the survey. There were eight question blocks:

1. Start: one question about how the invitation was received and one question about whether the respondent was currently working as an EV. If the answer to the second question was no, the participants could continue to answer the survey but were excluded from the study afterwards
2. Demographic information: nine questions about federal state, age, gender, children, professional experience, and other qualifications and memberships
3. Type of work: eight questions about leadership responsibilities, whether curative or not, animal species, size of facility, etc.
4. Working conditions: seven questions about working hours, overtime, and emergency services
5. Salary: nine questions about salary, vacation, additional benefits, and further training
6. Job satisfaction: one question with seven items. This question is taken from the German version of the Copenhagen Psychosocial Questionnaire III (COPSOQ, [23])
7. Miscellaneous: nine questions relating primarily to compliance with working time legislation
8. Educational background: four questions on parents’ educational background, previous vocational training, and admission procedures, and one open question for comments (results of this section are not part of this publication).

### 2.3. Data Management and Statistical Analyses

The data were exported directly from LimeSurvey to IBM SPSS (version 29.0.0.0, Armonk, NY, USA). First, data cleaning and management were carried out. Responses from individuals who stated they did not work as employed veterinarians, as well as those who completed fewer than 5 pages, were deleted. Moreover, responses from individuals who did not live in Germany were also excluded.

Age was calculated from the year of birth. Typing errors were corrected for two individuals (e.g., 1061 to 1961). Additional qualifications could be specified using multiple options, from which a new variable called “highest qualification” was formed for the multifactorial model. This included the following categories: “veterinary studies,” “doctorate or PhD,” “additional designation, master’s degree or GpCert, and other specific continuing education programs,” “national specialization, diplomate, and/or habilitation.” The variables gender and children were also further summarized for the model (female/male and children yes/no). When asked about the contractually fixed and actual working hours, 43 persons stated a range rather than a single number (e.g., 20–24 h). The mean value was assumed here. Eleven other data points contained implausible information (e.g., 9 for actual working hours, but 39 was specified for contractually agreed working hours). The responses of these individuals were deleted. The hourly wage was calculated from the monthly gross salary using the following formula:

hourly salary = (12 × monthly gross salary)/(52 × contractually fixed weekly working hours)

There were two implausible gross salary entries, which were deleted. Both the hourly salary for the gross salary without bonuses and with bonuses were calculated. If “0” was entered, the gross salary without bonuses was assumed as gross salary with bonuses. If individuals did not provide their gross salary, but only their net salary, this was estimated based on their tax bracket. This was the case for 38 individuals. The net-gross calculator on the handelsblatt.de website was used for this purpose, and the gross salary was calculated based on the tax bracket, federal state, and number of children. In addition to the gross salary calculated based on the contractually fixed working hours, the gross salary was also calculated based on the actual hours worked (including overtime).

A new variable called “section” was created based on whether the work was curative and the predominant type of animal treated. This included the following categories: non-curative, pets (including small rodents and exotic pets), horses, and farm animals (ruminants, pigs, poultry). Fifteen people had selected “other animal species” and provided further details in the free-text field. One person had specified “birds,” which was assigned to the “pets” category. Several people specified different animal species, which were assigned to the first-mentioned category. One person who stated that they treated primates was not assigned to any category.

Another variable called ‘corporate’ was created from the question “Is the institution you work for part of a group?” There were two categories here: a distinction was made between owner-managed practices (questions about the group answered with “no” or “yes,” and then VUK specified) and corporates (e.g., Anicura, Evidensia, Felmo, etc.).

Satisfaction was calculated from the seven satisfaction items according to the COPSOQ manual [23]. This can take values between 0 and 100, where higher values indicate greater satisfaction.

The data were primarily evaluated descriptively. The demographic data were compared with the 2024 veterinary statistics [14] to assess their representativeness. Comparisons between different groups—such as sections—were made using cross-tables and chi-square tests (CS; categorical variables). For quantitative dependent variables, such as satisfaction or hourly salary, either a Mann–Whitney U test (MWUT; for two groups) or a Kruskal–Wallis test (KWT; for more than two groups) was used, followed by a post hoc test with a Bonferroni correction for multiple comparisons.

To account for the influence of gender, children, full-time or part-time work, region, professional experience, additional qualifications, section, emergency service (yes/no), size of the facility, management responsibilities for other veterinarians, membership in the BaT or bpt (Bundesverband praktizierender Tierärzte e.V. [Association of practicing veterinarians]), and the number of overtime hours on the hourly wage, a multifactorial linear model was calculated. By including several variables in the same model, salary differences associated with, e.g., gender can be assessed independently of other factors that may also influence income, such as years in the profession or section of work. This reduces the risk that observed gender differences are explained solely by these other characteristics. Interactions were not examined. The endpoint was the hourly gross salary based on the contractually fixed working time with bonuses. We intended to perform backward selection to identify the best-performing model by selecting the model with the lowest Akaike’s Information Criterion (AIC) value. However, the model was not further reduced because removing the variable with the highest *p*-value increased the AIC slightly. Thus, the reduced model was not significantly better than the full model, so the full model was reported as the final model. The residuals were visually assessed for normality using a histogram. Despite the non-normal distribution of the target variable, the residuals were normally distributed.

For the comparison between owner-managed facilities and corporations, only individuals who worked in the pet and equine sectors were included. For logical reasons, this question was not asked of veterinarians in the non-curative sector, and veterinarians in the livestock sector were also excluded, as they would have, for example, skewed the satisfaction ratings for owner-managed practices upwards.

The significance level was set at *p* < 0.05. A reduced data set is provided as Supplementary Material File S3. The full data set cannot be provided, to protect respondents’ anonymity.

Grammarly (v1.2.260.1887, Grammarly Inc., San Francisco, CA, USA) was used for minor editing such as corrections of spelling, grammar, and to improve readability. No other generative AI was used in this study.

## 3. Results

### 3.1. Study Population, Demographics and Comparison to the Target Population

In total, 1519 data sets were exported from LimeSurvey. Responses from 320 people who had not answered at least page 5 of 8 were deleted. In addition, responses from 12 people who were not employed as EV and from three people who worked outside Germany were deleted. This left 1184 data sets, of which 1154 individuals completed the survey. However, the number of responses may still vary, as not every individual had to answer every question and, as explained, some of the questions were filter questions.

The demographics of the participants and the comparison with the target population (all veterinarians employed in Germany) are shown in Table 1. It can be seen that women were overrepresented in this survey. The federal states in which the survey was distributed by the chambers were overrepresented, while Hesse, Bavaria, and Baden-Württemberg, in particular, were underrepresented. The median age of the participants was 37 years. A quarter of the study population was 45 years or older, and a quarter was 31 years or younger. The participants had an average of 8 years of professional experience (median), with 25% of respondents having at least 16 years of professional experience. Just over half of the participants (54.6%, *n* = 642) had no children, 16.3% (*n* = 192) had one child; 22.5% (*n* = 264) had two children; and 6.6% (*n* = 77) had three or more children.

The majority of respondents had obtained their veterinary degree in Germany (90.8%, *n* = 1072). Ten respondents (0.8%) worked with a professional license, 13 respondents (1.1%) had retaken the exams, and 85 respondents (7.2%) had not obtained their veterinary degree in Germany, but their degree was recognized here. A good third of respondents had a doctorate (33.5%, *n* = 397), eleven had a PhD (0.9%), 52 had an additional qualification (4.4%), 116 had a national specialization (9.8%), ten had a diplomate title (0.8%), and three had obtained a habilitation (0.3%). Some 24.5% (*n* = 290) were members of the BaT; 20.0% of the participants (*n* = 237) were members of the Association of Practicing Veterinarians (bpt); and twelve people (1.0%) were members of the Federal Association of Public Veterinarians (Bundesverband der beamteten Tierärzte e.V.).

Some 28.1% of veterinarians working in a curative practice (296 of 1053) were employed by a corporation. Among those who mainly treated pets or horses, the proportion was just around one-third (33.0% and 33.1%, respectively), while the proportion among those who mainly treated farm animals was significantly lower (7.5%).

### 3.2. Working Hours, Overtime, and Emergency Service

**Figure 1:**
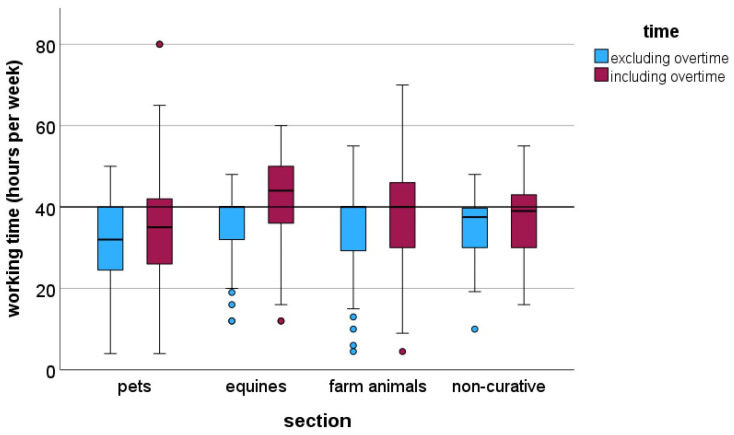
Weekly working of employed veterinarians with and without overtime by section from a survey in Germany (pets: *n* = 698; equines: *n* = 157; farm animals: *n* = 188; non-curative: *n* = 88).

The average contractually fixed working time was 35 h (median). Respondents working with horses worked significantly more hours than veterinarians in all other areas, and veterinarians working with farm animals worked significantly more than veterinarians working with pets (KWT: *p* < 0.001; Figure 1).

On average, respondents worked three hours of overtime per week. A quarter of respondents worked five or more hours of overtime per week. Although most hours are already worked contractually in the equine and farm animal sectors, the number of overtime hours was significantly higher there than in the pet sector (median: horses: 5, farm animals: 4, pets and non-curative: 2 h per week; KWT: *p* < 0.001). 30.1% of respondents stated they did not work overtime.

With regard to the handling of overtime, respondents could select multiple options: 17.4% of respondents (*n* = 206) stated that overtime expires; 38.2% (*n* = 452) stated that they receive time off in lieu as specified by their employer; 40.9% (*n* = 484) stated that they could take time off for overtime at their own discretion; 24.9% (*n* = 295) received a cash payment; and 32 people (*n* = 2.7%) received a bonus for overtime worked.

Around half of respondents (54.6%; *n* = 647) worked part-time (<38 h per week). Mothers were significantly more likely to work part-time than women without children (mothers: 82.5%, women without children: 38.4%, *p* < 0.001). This effect was not observed among men: here, 20.5% of fathers (9 of 44) worked part-time, while men without children were even more likely to be employed part-time (25.9%; 14 out of 54; *p* = 0.525).

**Figure 2:**
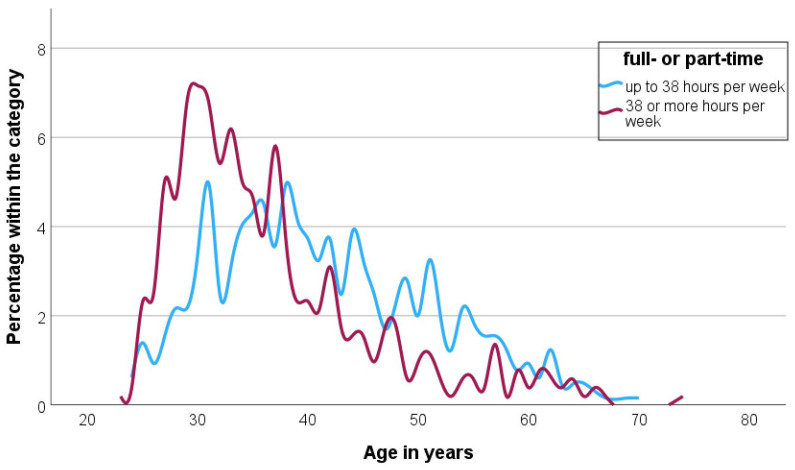
Distribution of full-time and part-time employees by age of the employed veterinarians surveyed (part-time (light blue): *n* = 646; full-time (dark red): *n* = 517).

Figure 2 shows the part-time rate for all genders by age. Up to the age of about 38 years, more respondents worked full-time than part-time. Among older participants, the proportion of part-time employees predominated. This does not change until the age of mid-60s.

Emergency services were provided by 75.2% of respondents (*n* = 875), with significant differences among sections: EV who mainly treated farm animals or equines almost always provided emergency services (farm animals: 89.5%; equines: 93.0%). Some 73.0% of EV who primarily treated pets provided emergency services. Only 28.1% of participants in the non-curative sector provided emergency services.

Of those who provided emergency services, more than half worked on one or two weekend days (68.0%, *n* = 570), but 8.9% of respondents also reported working four or more weekend days per month. Of those who were generally on emergency duty, 27.2% (*n* = 232) worked five or more nightshifts per month.

### 3.3. Compliance with the Working Hours Act

Table 2 shows the compliance with the provisions of the WHA. A total of 22.1% of respondents reported working more than 10 h at least once per week, and 40% reported not being able to take the minimum 30-min break. If it was agreed that violations occurred at least once a month or once a week, those affected were asked whether this bothered them. Regarding rest periods, about half of those affected reported that this bothered them (54.0%; 170 of 315). The situation was similar regarding exceeding the maximum working time (8 or 10 h; 371 of 699; 53.1%).

When asked whether their employer provides a system to record working hours, 73.4% (834 of 1137 respondents) answered that this was the case. A total of 186 respondents (16.4%) stated that this was not the case, and 30 people (2.6%) stated that they were required to work without documenting their hours. Some 41 people (3.6%) had the option but did not use it. This question included the option to provide further details in a free-text field. One person stated that time recording was carried out using a navigation device. Fourteen people stated that their employer did not provide a system, but that they documented their working hours themselves. Another fourteen people stated that the times were changed retrospectively to comply with the provisions of the WHA, that the system automatically clocked them out after ten hours, that it was not possible to work overtime, or that an hour’s break was automatically deducted, even if they had not taken it.

### 3.4. Salary

The average gross hourly salary with and without bonuses and with and without overtime is shown in Table 3 related to professional experience. It can be seen that veterinarians with more professional experience receive higher salaries and bonuses. When overtime is taken into account, the hourly salary is about €2 lower than the salary based on the agreed working time. When compared to the survey of 2020 [9], the hourly salary based on contractually fixed working with bonuses increased by 19%, adjusted for years of professional experience (File S4).

The results of the multifactorial model (Table 4) show that salaries rose significantly with increasing qualifications, professional experience, and leadership responsibilities. In addition, veterinarians in the north and west earned more than veterinarians in the east. Participants working in the equine section earned less, while those working in the non-curative section earned more compared to respondents in the pet section. Moreover, even when adjusted for these factors, women earned about 2 € less per hour than men, leading to an adjusted gender pay gap of about 7.4% related to the intercept. In contrast, being a member of professional associations, having children, participating in emergency services, the size of the facility, and part-time employment did not influence salary. The final model explained 39% of the variability of the salary (adjusted R-square).

### 3.5. Vacation and Training

Just under half of those surveyed (527 of 1152, 45.7%) had 30 days of vacation per year based on a 5-day work week. Just under 5% (4.6%, *n* = 53) had more vacation days, but 71 people (6.2%) reported having the legal maximum of 20 vacation days per year.

For just under half of the respondents (47.2%, 531 of 1126), further training was predominantly recognized as working time, while 478 people stated that they attended further training courses mainly in their free leisure time (42.5%). Just under 10% (9.8%, *n* = 110) usually took vacation days for further training, and seven people (0.6%) stated that they made up for the time. Regarding training costs, 11.9% of participants (141 of 1103) stated that their employer did not contribute any money, but 300 people (27.2%) stated that their employer would pay an average of more than €1000 for their training, including travel expenses.

### 3.6. Job Satisfaction

**Figure 3:**
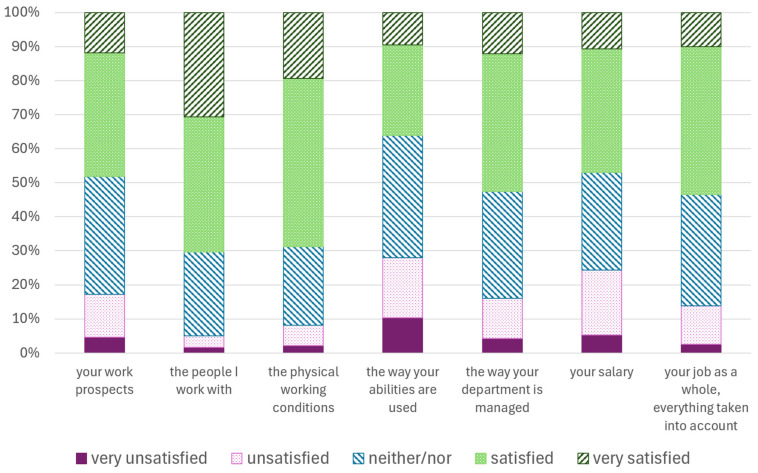
Agreement with the different items of job satisfaction from a study of employed veterinarians in Germany (*n* = 1157 to 1166).

The evaluation of the job satisfaction items is shown in Figure 3. Around 70% were (very) satisfied with their team, while satisfaction with the way their abilities are used, management, and salary was significantly lower.

**Figure 4:**
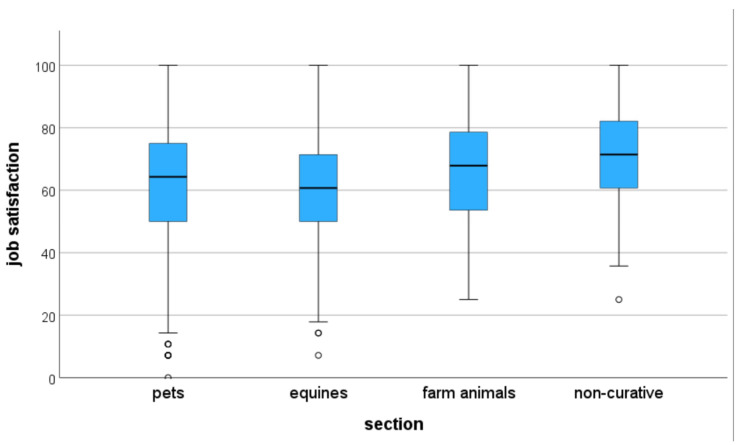
Satisfaction of employed veterinarians by section from a German survey (pets: *n* = 686, equines: *n* = 151, farm animals: *n* = 187, non-curative: *n* = 84).

The mean of all satisfaction items was 62.1 out of a possible 100 points. A comparison of the sections (Figure 4) showed that participants working in the non-curative sector were significantly more satisfied than participants working with equines or pets. In both cases, the adjusted significance was *p* < 0.001. Participants working with farm animals were significantly more satisfied than veterinarians working with equines (*p* = 0.030), while there was no significant difference between those working with pets and those working with equines (*p* > 0.999).

A total of 41.3% (458 of 1108) of respondents stated that their manager did not conduct an employee appraisal with them at least once a year. Those who had an appraisal with their manager were significantly more satisfied with the management (CQ: *p* < 0.001).

Some 882 of the respondents had a duty schedule. For 113 people (12.8%), this was set at least six months in advance, for 172 people (19.5%) at least three months in advance, and for 385 people (43.7%) at least one month in advance. A total of 212 people (24.0%) stated that the duty schedule was set less than one month in advance or that there were constant changes. Here, too, there was a significant association with the satisfaction with leadership: people whose duty schedule was only fixed less than one month in advance or was subject to constant changes were more likely to be very dissatisfied (*n* = 38, expected frequency: 22.2) or dissatisfied (*n* = 56, expected frequency: 37.5; CQ: *p* < 0.001).

Respondents with children were asked how they assessed the compatibility of work and family life. Of the 661 respondents, 5.1% rated it as “1—almost no problems,” 11.4% chose answer option 2, 41.7% chose option 3, 18.7% chose option 4, and 2.3% chose “5—disastrous.”

### 3.7. Comparison Between Employees of Corporate- and Owner-Managed Veterinary Practices

Employees of corporates worked significantly more overtime than people who were employed in an owner-managed practice (corporate: median = 3 h, owner-managed: 2 h; MWU: *p* < 0.001). There were no statistically significant differences between corporate- and owner-managed practices in terms of how overtime was handled. The average hourly salary (gross, including bonuses based on contractually fixed working hours) was significantly higher at corporate-owned than at owner-managed practices (median: corporations: €28.85; owner-managed: €27.87; *p* = 0.017). However, when the salary was based on actual hours worked, there was no significant difference (median: corporates: €25.96; owner-managed: €25.90; *p* = 0.139). Even in a multifactorial model that adjusted for region, gender, professional experience, highest additional qualification, and management responsibilities, there was no difference in hourly wages based on actual hours worked between owner-managed practices and practices owned by a corporate (*p* = 0.147).

Overall satisfaction was significantly higher among respondents working in owner-managed practices compared to those working for corporates (median: corporate: 57.1, owner-managed: 64.3; MWU: *p* < 0.001). In terms of the individual facets of satisfaction, employees of corporations were significantly more dissatisfied with career prospects (CQ: *p* = 0.006), physical working conditions (CQ: *p* = 0.001), salary (CQ: *p* < 0.001), and work overall (*p* = 0.017). There were no differences regarding the team and leadership. Employees of corporates had significantly more frequent employee appraisals at least once a year (CQ: *p* < 0.001). No significant differences were found between corporate-owned and owner-managed practices regarding the eleven-hour rest period (CQ: *p* = 0.190) and exceeding the maximum working time of ten hours (CQ: *p* = 0.073). However, employees of corporations were less likely to be able to take their breaks (CQ: *p* < 0.001).

## 4. Discussion

The survey provides detailed insight into the working conditions and salary structures of employed veterinarians in Germany. It shows that the provisions of the WHA continue to be disregarded. Furthermore, the results indicate that job satisfaction tends to be lower in corporate practices than in owner-managed practices.

### 4.1. Representativity and Limitations

As with all surveys using a convenience sample, response bias is to be expected here as well. A comparison with the target population (Table 1) showed that EV who work in curative medicine are significantly overrepresented. The results, therefore, primarily reflect the conditions and satisfaction of this group. In addition, women were overrepresented, as is often the case in surveys [4,9]. This may have led to an underestimation of salaries and an overestimation of the part-time rate. Even though some federal states were overrepresented and others underrepresented, this probably did not lead to any significant distortion, as this distortion is distributed rather randomly across Germany and only the southern region is generally somewhat underrepresented. Finally, this survey was significantly more successful than previous surveys at encouraging older veterinarians to participate [3,9]. While only 10% of participants in the first survey were older than 41 years [9], in this study, 25% were older than 45 years. For this reason, the authors decided not to report the median hourly salary, as it cannot be directly compared to the hourly salary from the first survey due to the higher level of professional experience.

Moreover, it should be noted that the comparison between corporate- and owner-managed facilities applies only to the sections of equine and small animal medicine. For livestock, no comparisons were made due to the small number of corporate employees. Additionally, all results are self-reported. While this is the most suitable and feasible method to assess job satisfaction, it might have introduced an information bias regarding salaries. We cannot rule out that in some cases, persons might, e.g., have reported their net income instead of their gross income. However, by giving the opportunity to report net income, we hope to have minimized this bias. Regarding other variables, such as gender, working conditions, or qualifications, we assume that the chance of information bias is low and that participants answered these questions honestly.

The full data set cannot be provided, to protect respondents’ anonymity. First, the data safety declaration stated that no personal information would be shared with any third parties. Second, gender, age, and other personal information could be used to identify individuals.

### 4.2. Job Satisfaction

Job satisfaction was measured using a validated instrument, allowing for a comparison with other populations. In this study, EV had a mean job satisfaction of 62 (of max. 100), which is comparable to the German reference population (mean 63 [23]) and slightly higher than in a recent study among German official veterinarians (mean 61 [24]). However, when comparing the results from this study with those from German studies among academics, particularly physicians, the latter reported significantly higher job satisfaction (means: academics 70 [25], dentists 75 [25], general practitioners 77–79 [26]). Moreover, a study on veterinary staff in Canada reported a significantly higher mean job satisfaction of 70 and 76 [27]. It should be noticed that the items used in this study might slightly differ from those used in the study from Canada [27]. Nevertheless, the findings indicate that the average job satisfaction of EV in Germany is rather low compared to other academic professions in Germany.

It is noteworthy that a considerable range of job satisfaction was observed in this study, particularly regarding satisfaction with leadership. This is consistent with earlier studies, including one from Canada [8,27]. It is precisely on this point that the comparison between corporate- and owner-managed institutions is particularly interesting: no significant differences were found here concerning leadership, in contrast to the study by Kogan et al. [22]. In the study by Kogan et al. [22], veterinarians working in owner-managed practices reported higher job satisfaction, even though those in corporate practices received more benefits, such as insurance or mental wellness programs. However, veterinarians in owner-managed practices felt more known as individuals and less pressure to generate revenue [22]. These findings align with the finding that employee appraisals were conducted significantly more frequently in corporate-managed practices. In owner-managed practices, the offer of employee appraisals was associated with higher job satisfaction regarding the management. This was not the case in corporates, leading to the assumption that these meetings are used more as performance reviews than as progress reviews. However, on the other hand, the low percentage of employees of owner-managed facilities who have employee appraisals indicates room for improvement.

Therefore, in general, corporates should strive to ensure that employees feel seen and valued as individuals, whereas owners of veterinary practices should implement standard leadership practices to improve EV’ commitment to the workplace.

### 4.3. Salaries and Gender Pay Gap

In addition to feedback, appreciation is reflected in salaries. The increase in the GOT has surely led to higher incomes and revenues for owners of veterinary practices and clinics. The salary of EV increased by 19% compared to the 2020 survey (9), adjusted for years of experience (File S4). For all German employees, salaries increased by around 23% during the same period [28]. Therefore, despite the increase of the GOT, EV’ salaries did not increase more than the average salary in Germany. Moreover, compared with similar professions, salaries were low: physicians in their qualification period earn around 42% more than EV in their first five years of employment [29]. Therefore, a further increase in salaries is needed to retain EV in their profession and attract further talents into this profession.

Worldwide, differences in income among genders have been noticed in the veterinary profession [3,30,31,32]. The determination of the gender pay gap is not easy, as female veterinarians tend to be younger and have less working experience and often work fewer hours compared to men. Therefore, we used the hourly salary as the endpoint and accounted for qualification, working experiences, and other factors by multifactorial modeling. In this study, an adjusted gender pay gap of 7% was observed. However, compared to the study of 2020 [9], where a 15% gap was observed, the gap has significantly declined.

### 4.4. Working Hours and Overtime

On the one hand, the veterinary profession sets high demands for availability during unusual working hours. On the other hand, the profession’s increasing feminization calls for more flexible solutions to help balance work and family life. This setting provides both chances and challenges. A recent study showed that veterinarians who frequently spent time with their families had a higher degree of mental well-being. Therefore, it is positive that the parents participating in this survey reported a mostly moderate to good balance between work and family. It is important to note that parents who left the veterinary field to care for children were not allowed to participate in this survey, and working conditions are a key reason why veterinarians leave the profession [33,34]. In this study, a high percentage of respondents whose children had probably grown up still worked part-time. Therefore, studies are needed to examine why veterinarians leave the profession or do not re-enter the profession after the family phase in full-time practice.

Comparing the percentage of part-time employees, it is noteworthy that mothers as well as fathers were more likely to work part-time than parents in other professions in Germany [35,36]. However, the percentage of men without children who worked part-time was also relatively high, while women without children were more likely to work full-time than the average in Germany [36].

A factor that might be linked to the high percentage of part-time positions is the amount of overtime. In Germany, around 11% of employees worked overtime [37]—in this study, nearly 70% stated that they did so. Since overtime is common, some veterinarians may choose to work part-time to reduce their actual working hours.

Due to the nature of the veterinary profession—unexpected events all around the clock—overtime and unusual working hours cannot be avoided. Nevertheless, enough workforce to meet these peaks in workload can reduce stress and demands [38]. Bonuses for work during nights and at the weekend might also compensate for the demands and increase job satisfaction.

Finally, violations of the WHA were regularly reported. However, only about 50% of respondents stated to be bothered thereby. Therefore, the flexibilization of this law should be pursued through collective agreement. Legal liability can thereby be ensured if employers and employees agree on longer and fewer shifts, for instance.

## 5. Conclusions

This study highlights various factors impacting EV salaries and job satisfaction. To ensure the future readiness and ongoing interest of EV and veterinary medicine students to work in curative practice, the following recommendations should be taken into consideration:

The 2022 GOT adjustment did not result in significantly higher salaries for EV than for other professions. The evaluation of the current version of the GOT will hopefully shed light on how the veterinary practice’s profit developed and why the increase did not result in higher salaries for EV. A further increase of the GOT, with regular adjustments to ensure EV’ salaries align with those of other academic professions, might be needed.

Flexibilization of working hours should be possible only through collective agreements, which would also end the gender pay gap. Until collective agreements are established, violations of the WHA should be reported and penalized to improve working conditions for EVs.

Leadership plays a crucial role in job satisfaction among EV. Employee appraisals should be introduced if they have not already been. Moreover, the planning of shifts and emergency services could be improved.

First, to reduce staff shortages, further studies are needed on the reasons for part-time work (especially among veterinarians outside/after the family phase). Second, we see a further need for research on the corporatization of veterinary practices and clinics. Here, qualitative and quantitative studies could: (a) explore reasons why veterinarians sell their business to corporates or start working there; (b) examine whether the associated expectations are met; (c) compare retention rates of owner- and corporate-managed practices; and (d) explore how corporate- and owner-managed practices differ concerning their business culture, room for negotiation, and the individual recognition of employees.

## Figures and Tables

**Table 1:** Demographic data and workplace characteristics from a survey of employed veterinarians in Germany and comparison with the target population according to veterinary statistics [14].

Item	*n* in Study Population	% in Study Population	% in Target Population
**Gender**			
Male	101	8.6	
Female	1073	91.2	78.8
Diverse	1	0.1	
**Federal State**			
Baden-Württemberg	57	4.9	9.9
Bavaria	102	8.7	19.0
Berlin	35	3.0	5.5
Brandenburg	68	5.8	3.6
Bremen	14	1.2	0.3
Hamburg	17	1.4	1.4
Hesse	29	2.5	7.2
Mecklenburg-Western Pomerania	5	0.4	2.1
Lower Saxony	134	11.4	14.9
North Rhine-Westphalia	391	33.3	17.1
Rhineland-Palatinate	20	1.7	4.4
Saarland	8	0.7	0.9
Saxony	117	10.0	4.8
Saxony-Anhalt	11	0.9	2.4
Schleswig-Holstein	130	11.1	4.7
Thuringia	37	3.1	1.9
**Section**			
None curative	91	7.9	42.5
Curative - thereof mainly:	1061	92.1	57.5
Pets	709	61.5	
Equines	159	13.8	
farm animal	193	16.8	
**Place of work**			
General practice	681	64.1	
Specialized practice	104	9.8	
Clinic	172	16.2	
University clinic	15	1.4	

Names of variables are displayed in bold.

**Table 2:** Compliance with the Working Hours Act from a survey among German employed veterinarians.

	No or Very Rarely *n* (%)	Yes, at Least Monthly *n* (%)	Yes, at Least Weekly *n* (%)
Does it happen that you are unable to take a 30-min break during a workday of at least 6 h?	348 (36.0)	236 (24.4)	384 (39.7)
Are you not able to rest for at least 11 h after a working day?	775 (70.3)	231 (20.9)	97 (8.8)
Do you work more than 10 h per day?	611 (54.7)	315 (28.2)	191 (17.1)

**Table 3:** Gross hourly salary (GHS; median) by professional experience based on data from a survey among employed veterinarians in Germany.

Years of Professional Experience	GHS Based on Contractually Fixed Working Hours Without Bonuses in € (*n*)	GHS Based on Contractually Fixed Working Hours with Bonuses in € (*n*)	GHS Based on Actual Working Hours with Bonuses in € (*n*)
0–1 year	21.35 (98)	21.39 (98)	19.89 (98)
2 years	23.08 (62)	23.18 (54)	20.88 (53)
3 years	23.37 (57)	23.22 (54)	21.65 (54)
4 years	25.64 (69)	26.42 (64)	23.08 (63)
5 years	25.96 (116)	27.69 (105)	24.73 (105)
6 years	25.96 (71)	28.61 (64)	25.96 (66)
7 years	28.37 (56)	29.42 (51)	26.77 (51)
8–9 years	28.60 (88)	29.76 (78)	28.01 (78)
10–11 years	29.42 (81)	30.29 (69)	28.85 (69)
12–13 years	29.41 (70)	30.65 (64)	29.04 (66)
14–15 years	31.68 (58)	31.73 (49)	28.96 (49)
16–19 years	31.07 (69)	31.82 (62)	29.74 (61)
20–24 years	34.23 (73)	34.62 (60)	30.83 (58)
25–29 years	33.32 (70)	36.62 (62)	31.21 (61)
≥30 years	34.61 (54)	36.06 (50)	35.70 (50)

**Table 4:** Factors influencing the hourly gross salary (based on contractually fixed working hours and including bonuses) of employed veterinarians. Data from a survey in Germany (*n* = 907).

	*n*	Estimate	LCI	UCI	*p*-Value
Intercept		26.51	24.41	28.61	<0.001
**Children**					0.087
Children	381	0.96	−0.14	2.05	
No children	526	Reference			
**Region**				global	0.022 *
South	133	−1.43	−2.92	0.06	0.060
East	223	−1.86	−3.12	−0.60	0.004 *
North	215	−0.52	−1.77	0.73	0.414
West	336	Reference			
**Part-time or full-time position (more or less than 38 h per week)**					0.724
Part-time	481	0.20	−0.91	1.31	
Full-time	426	Reference			
**Gender**					0.026 *
male	77	1.94	0.23	3.64	
female	830	Reference			
**Emergency service**					0.181
Not working in emergency service	218	−0.85	−2.10	0.40	
Working in emergency service	689	Reference			
**Section**					<0.001 *
Pets	545	Reference			
Equines	131	−1.53	−2.99	−0.07	0.040 *
Farm animal	160	−0.59	−1.92	0.74	0.742
Non-curative	71	5.07	3.12	7.02	<0.001 *
**Leadership position for other veterinarians**					<0.001 *
Yes	213	3.73	2.51	4.95	
No	694	Reference			
**Highest qualification**					<0.001 *
Approbation	564	Reference			
PhD/doctoral thesis	222	1.06	6.49	10.21	0.082
Further qualifications like MSc or else	41	3.44	1.12	5.76	0.004 *
National specialization/Diplomate/ habilitation	80	8.35	6.49	10.21	<0.001 *
**Membership in the Association of Practicing Veterinarians (bpt)**					0.087
No	718	Reference			
Yes	189	1.03	−0.15	2.20	
**Membership in the Association of Employed Veterinarians (BaT)**					0.564
No	673	Reference			
Yes	234	−0.32	−1.42	0.77	
**Size of facility (number of veterinary full-time positions)**					0.474
1–3	235	−0.95	−2.59	0.69	0.257
4–6	248	−0.46	−2.04	1.12	0.568
6–10	162	−1.17	−3.16	0.22	0.088
10–20	125	−0.81	−2.60	0.07	0.372
>20	137	Reference			
**Years of professional experience**		0.32	0.25	0.39	<0.001 *
**Overtime in hours**		0.03	−0.07	0.13	0.562

* Significant factors are marked in green and with asterisks. LCI: lower 95%-confidence interval. UCI: upper 95%-confidence interval. Names of variables are displayed in bold.

## Data Availability

The original contributions presented in this study are included in the Supplementary Material File S3. Further inquiries can be directed to the corresponding author(s).

## Supplementary Materials

The following supporting information can be downloaded at: https://www.mdpi.com/article/10.3390/vetsci13050494/s1, File S1: Questionnaire in the original language; File S2: Questionnaire in English; File S3: Data of this study. Due to privacy restrictions, sensitive data as gender or years in the profession cannot be shared; File S4: Comparison of the hourly gross salary based on contractually fixed working with bonuses adjusted for years of professional experience of the 2020 and 2025 study.

## Abbreviations

The following abbreviations are used in this manuscript:

BaT	Association of Employed Veterinarians [Bund angestellter Tierärzte e.V.]
bpt	Association of Practicing Veterinarians [Bundesverband Praktizierender Tierärzte e.V.]
COPSOQ	Copenhagen Psychosocial Questionnaire III
CS	Chi-square-test
EV	Employed veterinarians
FVE	Federation of Veterinarians in Europe
GOT	Fee Schedule for Veterinarians [Gebührenordnung für Tierärzte]
KWT	Kruskal-Wallis-test
MWUT	Mann-Whitney-U-test
VUK	Association of Owner-managed Small Animal Clinics [Verbund unabhängiger Kleintierkliniken e.V.]
WHA	Working Hours Act
