# Supplementary material for: Survey on the Working Conditions, Salary, and Job Satisfaction of Employed Veterinarians in Germany
Source: Vet Sci. 2026 May 19;13(5):494. doi: 10.3390/vetsci13050494 (PMC13211543; doi:10.3390/vetsci13050494)
Supplement: Supplementary file 1 [file vetsci-13-00494-s001.zip › S1_Questionnaire.pdf]

Arbeitsbedingungen und -zufriedenheit angestellter Tierärzt:innen

Liebe Kolleg:innen,

wir möchten erfahren, wie es Ihnen in Ihrem Beruf geht, welche Leistungen Sie erhalten und was Sie dafür leisten. Bitte nehmen Sie sich die Zeit und füllen Sie die Umfrage möglichst vollständig aus. Sie schaffen damit eine wichtige berufspolitische und wissenschafltiche Diskussionsgrundlage!

Die Teilnahme erfolgt selbstverständlich anonym und Sie können sie jederzeit ohne Konsequenzen beenden. Es dauert etwa 10 bis 15 Minuten die Fragen zu beantworten.

Herzliche Grüße und vielen Dank,

Der Bund angestellter Tierärzte e.V.

In dieser Umfrage sind 50 Fragen enthalten.

Start

Wie sind Sie auf diese Umfrage aufmerksam geworden?

Bitte wählen Sie eine der folgenden Antworten:
Bitte wählen Sie nur eine der folgenden Antworten aus:

- ◯ Soziale Medien
- ◯ BaT-Newsletter
- ◯ Konferenz/ Tagung
- ◯ Website
- ◯ Printmedien
- ◯ Freund:innen oder Kolleg:innen
- ◯ Sonstiges ________

Sind Sie derzeit als Tierärztin/ Tierarzt angestellt? *

Bitte wählen Sie eine der folgenden Antworten:
Bitte wählen Sie nur eine der folgenden Antworten aus:

- ◯ Ja
- ◯ Ja, aber ich arbeite derzeit nicht (bspw. Mutterschutz)
- ◯ Nein

Wenn Sie "Nein" wählen, können Sie die Umfrage bis zum Ende sehen/ beantworten. Ihre Antworten werden aber nicht bei der Auswertung berücksichtigt.

Demografische Angaben

In welchem Jahr sind Sie geboren? *

In dieses Feld dürfen nur Zahlen eingegeben werden.
Bitte geben Sie Ihre Antwort hier ein:

____

In welchem Bundesland arbeiten Sie (überwiegend)?

Bitte wählen Sie eine der folgenden Antworten:
Bitte wählen Sie nur eine der folgenden Antworten aus:

- ◯ Baden-Württemberg
- ◯ Bayern
- ◯ Berlin
- ◯ Brandenburg
- ◯ Bremen
- ◯ Hamburg
- ◯ Hessen
- ◯ Mecklenburg-Vorpommern
- ◯ Niedersachsen
- ◯ Nordrhein-Westfalen
- ◯ Rheinland-Pfalz
- ◯ Saarland
- ◯ Sachsen
- ◯ Sachsen-Anhalt
- ◯ Schleswig-Holstein
- ◯ Thüringen
- ◯ außerhalb von Deutschland

Welches Geschlecht haben Sie?

Bitte wählen Sie eine der folgenden Antworten:
Bitte wählen Sie nur eine der folgenden Antworten aus:

- ◯ weiblich
- ◯ männlich
- ◯ divers
- ◯ möchte ich nicht angeben

Haben Sie Kinder?

Bitte wählen Sie eine der folgenden Antworten:
Bitte wählen Sie nur eine der folgenden Antworten aus:

- ◯ nein
- ◯ ja, ein Kind
- ◯ ja, zwei Kinder
- ◯ ja, drei oder mehr Kinder

Wie erleben Sie die Vereinbarkeit von Familie und Beruf?

Beantworten Sie diese Frage nur, wenn folgende Bedingungen erfüllt sind:
G01Q04 == 1 OR G01Q04 == 2 OR G01Q04 == 3

Bitte wählen Sie eine der folgenden Antworten:
Bitte wählen Sie nur eine der folgenden Antworten aus:

- ◯ 1 - nahezu problemlos
- ◯ 2
- ◯ 3
- ◯ 4
- ◯ 5 - katastrophal

Haben Sie Ihr Tiermedizinstudium in Deutschland abgeschlossen? *

Bitte wählen Sie eine der folgenden Antworten:
Bitte wählen Sie nur eine der folgenden Antworten aus:

- ◯ Ja
- ◯ Nein, aber mein Abschluss wird hier anerkannt (EU)
- ◯ Nein, ich habe die Prüfungen nachgeholt
- ◯ Nein, ich arbeite mit Berufserlaubnis

Wie viele Jahre arbeiten Sie bereits als Tierarzt/ Tierärztin? *

In dieses Feld dürfen nur Zahlen eingegeben werden.
Bitte geben Sie Ihre Antwort hier ein:

Welche zusätzlichen Qualifikationen haben Sie?

Kommentieren wenn eine Antwort gewählt wird

Bitte wählen Sie die zutreffenden Punkte aus und schreiben Sie einen Kommentar dazu:

- [ ] Promotion
- [ ] PhD
- [ ] Zusatzbezeichnung
- [ ] Fachtierarzt
- [ ] Diplomate
- [ ] Habilitation

Sonstiges:

In welchen Vereinen sind Sie Mitglied?

Wählen Sie alle zutreffenden Optionen

Bitte wählen Sie alle zutreffenden Antworten aus:

- [ ] Bundesverband praktizierender Tierärzte (bpt)
- [ ] Bund angestellter Tierärzte (BaT)
- [ ] Bundesverband der beamteten Tierärzte (bbt)

Tätigkeit

Haben Sie Führungsaufgaben für andere Tierärzt:innen?

Bitte wählen Sie eine der folgenden Antworten:
Bitte wählen Sie nur eine der folgenden Antworten aus:

- ◯ Ja
- ◯ Nein

Bitte schreiben Sie einen Kommentar zu Ihrer Auswahl

Arbeiten Sie überwiegend im kurativen Bereich?

Bitte wählen Sie eine der folgenden Antworten:
Bitte wählen Sie nur eine der folgenden Antworten aus:

- ◯ Ja
- ◯ Nein

In welcher Branche sind Sie tätig?

Beantworten Sie diese Frage nur, wenn folgende Bedingungen erfüllt sind:
G02Q02 == 2

Bitte wählen Sie eine der folgenden Antworten:
Bitte wählen Sie nur eine der folgenden Antworten aus:

- ◯ Öffentliches Veterinärwesen
- ◯ Industrie
- ◯ Hochschule

Bitte schreiben Sie einen Kommentar zu Ihrer Auswahl

Welche Tierarten behandeln Sie überwiegend?

Beantworten Sie diese Frage nur, wenn folgende Bedingungen erfüllt sind:
G02Q02 == 1

Bitte wählen Sie eine der folgenden Antworten:
Bitte wählen Sie nur eine der folgenden Antworten aus:

- ◯ Kleintiere
- ◯ Heimtiere
- ◯ Pferde
- ◯ Wiederkäuer
- ◯ Schweine
- ◯ Geflügel
- ◯ Fische
- ◯ Exoten
- ◯ Sonstiges ________

Welchen Status hat die Einrichtung, für die Sie arbeiten?

Beantworten Sie diese Frage nur, wenn folgende Bedingungen erfüllt sind:
G02Q02 == 1

Bitte wählen Sie eine der folgenden Antworten:
Bitte wählen Sie nur eine der folgenden Antworten aus:

- ◯ Klinik
- ◯ Tiergesundheitszentrum
- ◯ Praxis
- ◯ spezialisierte Praxis/ Überweisungspraxis
- ◯ Uni-Klinik

Ist die Einrichtung, für die Sie tätig sind, Teil einer Gruppe?

Beantworten Sie diese Frage nur, wenn folgende Bedingungen erfüllt sind:
G02Q02 == 1

Bitte wählen Sie eine der folgenden Antworten:
Bitte wählen Sie nur eine der folgenden Antworten aus:

- ◯ Ja
- ◯ Nein

Welcher Gruppe gehört die Einrichtung an?

Beantworten Sie diese Frage nur, wenn folgende Bedingungen erfüllt sind:
G02Q02 == 1 AND G02Q06 == 1

Bitte wählen Sie eine der folgenden Antworten:
Bitte wählen Sie nur eine der folgenden Antworten aus:

- ◯ Anicura
- ◯ Evidensia
- ◯ Tierarzt Plus Partner
- ◯ Smartemis
- ◯ VUK
- ◯ Filu
- ◯ Rex
- ◯ felmo
- ◯ Altano
- ◯ Veternicum
- ◯ Sonstiges ______

Wie viele Tierärzt:innen arbeiten in Ihrer Einrichtung (Teilzeitkräfte anteilig)?

Bitte wählen Sie eine der folgenden Antworten:
Bitte wählen Sie nur eine der folgenden Antworten aus:

- ◯ 1-3
- ◯ 4-6
- ◯ 6-10
- ◯ 10-20
- ◯ mehr als 20

Arbeitsbedingungen

Wie viele Stunden arbeiten Sie laut Arbeitsvertrag pro Woche? *

Bitte geben Sie Ihre Antwort hier ein:

Wie viele Stunden arbeiten Sie pro Woche tatsächlich im Durchschnitt? *

Bitte geben Sie Ihre Antwort hier ein:

Wie wird mit den Überstunden umgegangen?

Wählen Sie alle zutreffenden Optionen
Bitte wählen Sie alle zutreffenden Antworten aus:

- [ ] Ich mache keine Überstunden
- [ ] Freizeitausgleich (Führungskraft entscheidet wann)
- [ ] Abbummeln (ich entscheide wann)
- [ ] Auszahlung
- [ ] Zuschlag
- [ ] Verfallen

Leisten Sie Notdienste?

Bitte wählen Sie eine der folgenden Antworten:
Bitte wählen Sie nur eine der folgenden Antworten aus:

- ○ Ja
- ○ Nein

Notdienst = Untersuchung und Behandlung außerhalb der Sprechzeiten

Wie häufig pro Monat leisten Sie durchschnittlich Nachtdienste (inkl. Rufbereitschaft)?

Beantworten Sie diese Frage nur, wenn folgende Bedingungen erfüllt sind:
G03Q04 == 1

Bitte wählen Sie eine der folgenden Antworten:
Bitte wählen Sie nur eine der folgenden Antworten aus:

- ◯ keine
- ◯ 1
- ◯ 2
- ◯ 3
- ◯ 4
- ◯ 5
- ◯ 6
- ◯ 7 oder mehr

An wie vielen Tagen pro Monat leisten Sie durchschnittlich Wochenend-Dienste (reguläre Sprechstunde samstags ausgenommen)?

Beantworten Sie diese Frage nur, wenn folgende Bedingungen erfüllt sind:
G03Q04 == 1

Bitte wählen Sie eine der folgenden Antworten:
Bitte wählen Sie nur eine der folgenden Antworten aus:

- ◯ 0
- ◯ 1
- ◯ 2
- ◯ 3
- ◯ 4
- ◯ 5
- ◯ 6 oder mehr

Erhalten Sie Zuschläge für Nacht- oder Wochenenddienste?

Beantworten Sie diese Frage nur, wenn folgende Bedingungen erfüllt sind:
G03Q04 == 1

Bitte wählen Sie eine der folgenden Antworten:
Bitte wählen Sie nur eine der folgenden Antworten aus:

- ◯ Nein
- ◯ Ja, für Nachtdienste
- ◯ Ja, für Wochenenddienste
- ◯ Ja, sowohl für Nacht- als auch Wochenenddienste

Gehalt

Wie hoch ist Ihr monatliches Brutto-Grundgehalt ohne Zuschläge? *

In dieses Feld dürfen nur Zahlen eingegeben werden.
Bitte geben Sie Ihre Antwort hier ein:

[ ]

Bitte schauen Sie Ihr Gehalt - wenn Sie es nicht wissen - auf Ihrer Lohnabrechnung nach. Sollten Sie diese nicht zur Hand haben, tragen Sie bitte 0 ein.

Wie hoch ist Ihr monatliches Brutto-Gehalt mit Zuschlägen?

In dieses Feld dürfen nur Zahlen eingegeben werden.
Bitte geben Sie Ihre Antwort hier ein:

[ ]

Bitte schauen Sie Ihr Gehalt - wenn Sie es nicht wissen - auf Ihrer Lohnabrechnung nach. Sollten Sie diese nicht zur Hand haben, tragen Sie bitte 0 ein.

Wie hoch ist Ihr monatliches **Netto**-Gehalt (Auszahlungsbetrag)?

Beantworten Sie diese Frage nur, wenn folgende Bedingungen erfüllt sind:
G04Q01 == 0 OR G04Q02 == 0

Bitte geben Sie Ihre Antwort hier ein:

In welcher Lohnsteuerklasse sind Sie?

Beantworten Sie diese Frage nur, wenn folgende Bedingungen erfüllt sind:
G04Q03 > 0

Bitte wählen Sie eine der folgenden Antworten:
Bitte wählen Sie nur eine der folgenden Antworten aus:

- ◯ I
- ◯ II
- ◯ III
- ◯ IV
- ◯ V
- ◯ VI

Falls Sie schon seit November 2022 mit der gleichen Stundenzahl bei Ihrem derzeitigen Arbeitgeber angestellt sind: Um wie viel € hat sich Ihr Brutto-Gehalt seitdem erhöht?

Beantworten Sie diese Frage nur, wenn folgende Bedingungen erfüllt sind:
G01Q06 > 2

Bitte geben Sie Ihre Antwort hier ein:

[ ]

Falls keine Erhöhung: bitte 0 eintragen

Falls Sie noch nicht so lange dort tätig sind: bitte nichts eintragen

Falls Sie es nicht wissen: ? eintragen

Welche Zusatzleistungen erhalten Sie?

Wählen Sie alle zutreffenden Optionen
Bitte wählen Sie alle zutreffenden Antworten aus:

- [ ] 13. Monatsgehalt
- [ ] Vermögenswirksame Leistungen
- [ ] Betriebliche Altersvorsorge
- [ ] Weihnachtsgeld
- [ ] Umsatzbeteiligung
- [ ] Dienstwagen zur privaten Nutzung
- [ ] Sachleistungen oder Gutscheine
- [ ] Kinderbetreuungskosten
- [ ] Gesundheitsleistungen (Fitness, Massage, ...)
- [ ] kostenloses Essen
- [ ] kostenlose /vergünstigte Behandlung des eigenen Tiers
- [ ] Urlaubsgeld
- [ ] Sonstiges: [ ]

Wie viele Urlaubstage pro Jahr haben Sie (bezogen auf eine 5-Tage-Woche)?

In dieses Feld dürfen nur Zahlen eingegeben werden.
Bitte geben Sie Ihre Antwort hier ein:

Wie wird bei Ihnen überwiegend mit Fortbildungen verfahren?

Bitte wählen Sie eine der folgenden Antworten:
Bitte wählen Sie nur eine der folgenden Antworten aus:

- Ich muss mir Urlaub nehmen
- Ich mache Fortbildungen in der Freizeit
- Ich muss die Zeit nacharbeiten
- Fortbildungen werden als Arbeitszeit anerkannt

Wie viel Geld zahlt Ihr Arbeitgeber jährlich für Ihre Fortbildungen (inkl. Reisenkosten; durchschnittlich über die letzten Jahre)?

Bitte wählen Sie eine der folgenden Antworten:
Bitte wählen Sie nur eine der folgenden Antworten aus:

- Er steuert kein Geld bei
- bis etwa 250 €
- bis etwa 500 €
- bis etwa 1000 €
- mehr als 1000 €

Arbeitszufriedenheit

Wenn Sie Ihre Arbeitssituation insgesamt betrachten, wie zufrieden sind Sie mit...

Bitte wählen Sie die zutreffende Antwort für jeden Punkt aus:

	sehr zufrieden	zufrieden	teils-teils	unzufrieden	sehr unzufrieden
... Ihren Berufsperspektiven?	○	○	○	○	○
... den Leuten, mit denen Sie arbeiten?	○	○	○	○	○
... den körperlichen Arbeitsbedingungen?	○	○	○	○	○
... der Art und Weise, wie Ihre Abteilung geführt wird?	○	○	○	○	○
... der Art und Weise, wie Ihre Fähigkeiten genutzt werden?	○	○	○	○	○
... Ihrem Gehalt?	○	○	○	○	○
... Ihrer Arbeit insgesamt unter Berücksichtigung aller Umstände?	○	○	○	○	○

Sonstiges

Führt Ihre Führungskraft mindestens einmal jährlich ein Mitarbeitergespräch mit Ihnen?

Bitte wählen Sie eine der folgenden Antworten:
Bitte wählen Sie nur eine der folgenden Antworten aus:

- ◯ Ja
- ◯ Nein

Wann steht für Sie der Dienstplan fest?

Bitte wählen Sie eine der folgenden Antworten:
Bitte wählen Sie nur eine der folgenden Antworten aus:

- ◯ es gibt keinen Dienstplan
- ◯ weniger als einen Monat zuvor oder es kommt ständig zu Änderungen
- ◯ mindestens einen Monat im Voraus
- ◯ mindestens drei Monate im Voraus
- ◯ mindestens sechs Monate im Voraus

Sorgt Ihr Arbeitgeber dafür, dass Sie Ihre Arbeitszeit erfassen (können)?

Bitte wählen Sie eine der folgenden Antworten:
Bitte wählen Sie nur eine der folgenden Antworten aus:

- ◯ Ja und ich erfasse sie relativ genau
- ◯ Ja, aber ich mache das nicht
- ◯ Ja, aber ich bin angehalten zu arbeiten ohne dies zu dokumentieren
- ◯ Nein, es gibt keine Arbeitszeiterfassung
- ◯ Sonstiges ____________

Können Sie mindestens 30 Minuten Pause machen?

Bitte wählen Sie eine der folgenden Antworten:
Bitte wählen Sie nur eine der folgenden Antworten aus:

- ◯ Ich arbeite max. 6 Stunden täglich und brauche keine Pause
- ◯ Ja, das geht immer
- ◯ Mindestens einmal im Monat klappt das nicht
- ◯ Mindestens einmal pro Woche klappt das nicht

Haben Sie nach einem Arbeitstag mindestens 11 Stunden arbeitsfreie Zeit?

Bitte wählen Sie eine der folgenden Antworten:
Bitte wählen Sie nur eine der folgenden Antworten aus:

- ◯ Ja, eigentlich (fast) immer
- ◯ Mindestens einmal pro Monat klappt das nicht
- ◯ Mindestens einmal pro Woche wird die Ruhezeit unterschritten

Stört Sie das?

Beantworten Sie diese Frage nur, wenn folgende Bedingungen erfüllt sind:
G06Q05 == 2 Or G06Q05 == 3

Bitte wählen Sie eine der folgenden Antworten:
Bitte wählen Sie nur eine der folgenden Antworten aus:

- ◯ Nein
- ◯ Ja

Kommt es vor, dass Sie mehr als acht Stunden pro Tag arbeiten?

Bitte wählen Sie eine der folgenden Antworten:
Bitte wählen Sie nur eine der folgenden Antworten aus:

- ◯ Nein, nur sehr selten
- ◯ Nur maximal einmal pro Monat
- ◯ Ja, mindestens einmal pro Woche

Kommt es vor, dass Sie mehr als **zehn** Stunden an einem Tag arbeiten?

Beantworten Sie diese Frage nur, wenn folgende Bedingungen erfüllt sind:
G06Q06 == 2 OR G06Q06 == 3

Bitte wählen Sie eine der folgenden Antworten:
Bitte wählen Sie nur eine der folgenden Antworten aus:

- ◯ Nein oder nur sehr selten
- ◯ Mindestens einmal pro Monat
- ◯ Ja, mindestens einmal pro Woche

Stört Sie die Überschreitung der täglichen Höchstarbeitsdauer?

Beantworten Sie diese Frage nur, wenn folgende Bedingungen erfüllt sind:
G06Q06 == 3 OR G06Q07 == 2 OR G06Q07 == 3

Bitte wählen Sie eine der folgenden Antworten:
Bitte wählen Sie nur eine der folgenden Antworten aus:

- ◯ Ja
- ◯ Nein

Bildungsbiografie

Welche Aussage trifft auf Sie am ehesten zu?

Bitte wählen Sie eine der folgenden Antworten:
Bitte wählen Sie nur eine der folgenden Antworten aus:

- ◯ Ich wollte schon als Kind Tierarzt/ Tierärztin werden
- ◯ Der Berufswunsch kam erst später
- ◯ Eigentlich hätte ich mir auch gut einen anderen Beruf vorstellen können
- ◯ Der Wunsch, dass ich Tierarzt/ Tierärztin werde, stammte von jemand anderem

Hat mindestens ein Elternteil von Ihnen ein Hochschulstudium absolviert?

Bitte wählen Sie eine der folgenden Antworten:
Bitte wählen Sie nur eine der folgenden Antworten aus:

- ◯ Ja
- ◯ Nein

Haben Sie vor dem Studium eine Berufsausbildung absolviert?

Bitte wählen Sie eine der folgenden Antworten:
Bitte wählen Sie nur eine der folgenden Antworten aus:

- ◯ Ja, in einem tiermedizinischen Bereich (z.B.: TFA, Landwirtschaft)
- ◯ Ja, in einem anderen Bereich
- ◯ Nein

Über welches Auswahlverfahren wurden Sie zum Studium zugelassen?

Bitte wählen Sie eine der folgenden Antworten:
Bitte wählen Sie nur eine der folgenden Antworten aus:

- ◯ Abi-Bestnote
- ◯ Auswahlverfahren der Hochschule (TMS, Abi, Motivationstest etc.)
- ◯ Vorab-Quote (Härtefall, Ausländerquote, Zweitstudium, ...)
- ◯ Wartezeit
- ◯ Nachrückverfahren
- ◯ Losverfahren
- ◯ weiß nicht
- ◯ Sonstiges ________

Haben Sie noch Anmerkungen/ Kommentare, die Sie uns mitteilen möchten?

Bitte geben Sie Ihre Antwort hier ein:

Herzlichen Dank für Ihre Teilnahme!

Wenn Sie am Gewinnspiel teilnehmen möchten, dann klicken Sie bitte auf den folgenden Link und geben Sie Ihre Email-Adresse ein. Diese wird getrennt von den eben übermittelten Daten gespeichert und kann nicht mit diesen in Verbindung gebracht werden.

https://vetepi.limesurvey.net/754494?lang=de

https://bundangestelltertieraerzte.de/jetzt-mitglied-im-bat-werden/

08.12.2025 – 11:45

Senden Sie Ihre Umfrage ein.

Vielen Dank für die Beantwortung des Fragebogens.
