# Supplementary material for: Survey on the Working Conditions, Salary, and Job Satisfaction of Employed Veterinarians in Germany
Source: Vet Sci. 2026 May 19;13(5):494. doi: 10.3390/vetsci13050494 (PMC13211543; doi:10.3390/vetsci13050494)
Supplement: Supplementary file 1 [file vetsci-13-00494-s001.zip › S2_questionnaire english.pdf]

Working Conditions and Job Satisfaction Among Employed Veterinarians

Dear colleagues,

We would like to learn how you are faring in your profession, what benefits you receive, and what you do in return. Please take the time to complete the survey as thoroughly as possible. By doing so, you will help create an important foundation for professional and scientific discussion!

Participation is, of course, anonymous, and you can withdraw at any time without any consequences. It takes about 10 to 15 minutes to answer the questions.

Best regards and thank you very much,

The Association of Employed Veterinarians

This survey contains 50 questions.

Start

How did you hear about this survey?

Social media

BaT newsletter

Conference/meeting

Website

Print media

Friends or colleagues

Other: ____________________

Are you currently employed as a veterinarian?*

* If you select "No," you can view/answer the survey until the end. However, your answers will not be included in the analysis.

Yes

Yes, but I am not currently working (e.g., maternity leave)

No

What year were you born?

Only numbers may be entered in this field.

Please enter your answer here: ___________

In which state do you work (primarily)?

Please select one of the following answers:

Please select only one of the following answers:

Baden-Württemberg

Bavaria

Berlin

Brandenburg

Bremen

Hamburg

Hesse

Mecklenburg-Western Pomerania

Lower Saxony

North Rhine-

Westphalia

Rhineland-Palatinate

Saarland

Saxony

Saxony-Anhalt

Schleswig-Holstein

Thuringia

outside Germany

What is your gender?

female

male

non-binary

I prefer not to say

Do you have children?

No

Yes, one child

Yes, two children

Yes, three or more children

If children=yes

How do you manage balancing family and work?

1 - almost no problems

2

3

4

5 – disastrous

Did you complete your veterinary degree in Germany?

Yes

No, but my degree is recognized here (EU)

No, I took the exams to make up for them

No, I am working with a professional license

How many years have you been working as a veterinarian?

Only numbers may be entered in this field.

Please enter your answer here: ___________

What additional qualifications do you have?

Please select the applicable items and write a comment:

Doctoral thesis: ________________

PhD: ________________

Zusatzbezeichnung (national qualification): ___________________

Fachtierarzt (national qualification): ________________

Diplomate: ________________

Habilitation: ________________

Other: _________________

Which organizations are you a member of?

Association of Practicing Veterinarians (bpt)

Association of Employed Veterinarians (BaT)

Federal Association of Public Veterinarians (bbt)

Do you have management responsibilities for other veterinarians?

Yes

No

Do you primarily work in the curative field?

Yes

No

If not curative:

In which section do you work?

Public veterinary services

Industry

Higher education

If curative:

Which animal species do you primarily treat?

Pets

Small mammals

Horses

Ruminants

Pigs

Poultry

Fish

Exotic animals

Other

If curative:

What is the status of the institution you work for?

Veterinary clinic

Animal health center

General practice

Specialized practice

University hospital

If curative:

Is the facility where you work part of a group?

Yes

No

If curative and if group=yes

Which group does the facility belong to?

Anicura

Evidensia

Veterinarian Plus

Partner Smartemis

VUK

Filu

Rex

Felmo

Altano

Veternicum

Other: ______________________

How many veterinarians work at your facility (part-time staff included on a pro-rata basis)?

1-3

4-6

6-10

10-20

more than 20

Working conditions

How many hours per week do you work according to your employment contract?

____

How many hours do you actually work on average per week?

____

How is overtime handled?

I don’t work overtime

Time off in lieu (manager decides when)

Time off in lieu (I decide when)

Payment

bonus

expires

Do you participate in emegency services?

Yes

No

Emergency service = Examination and treatment outside of office hours

If emergency service=yes

On average, how many night shifts (including on-call duty) do you work per month?

None

1

2

3

4

5

6

7 or more

If emergency service=yes

On average, how many days per month do you work on weekends (excluding regular opening hours on Saturdays)?

0

1

2

3

4

5

6 or more

If emergency service=yes

Do you receive bonuses for night or weekend shifts?

No

Yes, for night shifts

Yes, for weekend shifts

Yes, for both night and weekend shifts

Salary

What is your monthly gross base salary without bonuses? *

Only numbers may be entered in this field.

Please enter your answer here: ____________

If you don't know your salary, please check your pay stub. If you don't have it on hand, please enter 0

What is your monthly gross salary including bonuses?

Only numbers may be entered in this field.

Please enter your answer here: ____

Please check your pay stub for your salary if you don’t know it. If you don’t have it on hand, please enter 0.

If 0 was entered for gross salary:

What is your monthly net salary (pay amount)?

Please enter your answer here: ___________

If 0 was entered for gross salary:

What is your income tax bracket?

I

II

III

IV

V

VI

If you have been employed by your current employer with the same number of hours since November 2022: By how many euros has your gross salary increased since then?

Please enter your answer here: ______________

If there was no increase: please enter 0

If you have not been working there that long: please enter nothing

If you do not know: enter ?

What additional benefits do you receive?

Select all applicable options

13th month’s salary

Capital-forming benefits

Company pension plan

Christmas bonus

Profit sharing

Company car for personal use

Benefits in kind or vouchers

Childcare costs

Health benefits (fitness, massage, ...)

Free meals

Free/discounted veterinary care for your own pet

Vacation pay

Other: _________________

How many vacation days do you have per year (based on a 5-day workweek)?

Only numbers may be entered in this field.

Please enter your answer here: ___________

How is continuing education generally handled at your organization?

I have to take vacation time

I do continuing education in my free time

I have to make up the time

Continuing education is recognized as work time

How much does your employer pay annually for your training (including travel expenses; average over the last few years)?

They do not contribute any money

up to about€250

up to about €500

up to about €1,000

more than €1,000

Job satisfaction

When you look at your overall work situation, how satisfied are you with...

Answer options: very unsatisfied; unsatisfied; neither/nor; satisfied; very satisfied

... your career prospects?

... the people you work with?

... the physical working conditions?

... the way your department is managed?

... the way your skills are used?

... your salary?

... your job overall, taking all circumstances into account?

Other

Does your manager conduct an employee appraisal with you at least once a year?

Yes

No

When is your work schedule finalized?

There is no work schedule

Less than a month in advance, or there are constant changes

At least one month in advance

At least three months in advance

At least six months in advance

Does your employer ensure that you (can) track your working hours?

Yes, and I track them fairly accurately

Yes, but I don’t do that

Yes, but I am required to work without documenting it

No, there is no time tracking

Other: ________________________

Can you take a break for at least 30 minutes?

I work a maximum of 6 hours a day and don’t need a break

Yes, that’s always possible

At least once a month, that doesn’t work out

At least once a week, that doesn’t work out

Do you have at least 11 hours of time off after a workday?

Yes, actually (almost) always

At least once a month, that doesn’t work out

At least once a week, I don’t get that much rest time

If rest time=at least…

Does this bother you?

No

Yes

Do you ever work more than eight hours a day?

No, only very rarely

At most once a month

Yes, at least once a week

If more than eight hours= once a month or at least once a week

Do you ever work more than ten hours a day?

No, only very rarely

At least once a month

Yes, at least once a week

If eight hours=at least weekly or ten hours=monthly or weekly:

Does exceeding the maximum daily working hours bother you?

Yes

No

Educational Background

Which statement best applies to you?

I wanted to be a veterinarian even as a child

The career choice came later

Actually, I could have easily imagined myself in a different profession

The idea of me becoming a veterinarian came from someone else

Did at least one of your parents complete a college degree?

Yes

No

Did you complete vocational training before starting your studies?

Yes, in a veterinary field (e.g., veterinary technician, agriculture)

Yes, in another field

No

Through which admissions process were you admitted to the program?

Highest Abitur grade

University selection process (TMS, high school diploma,

motivation test, etc.)

Priority quota (hardship cases, internationalstudent quota, second degree, ...)

Waiting list (waiting several semesters)

Waiting list (because someone else did not take the place)

lottery

Don’t know

Other: ____________________

Do you have any additional comments or remarks you’d like to share with us?

Thank you very much for participating!

If you would like to enter the contest, please click on the following link and enter your email address. This will be stored separately from the data you just submitted and cannot be linked to it.

https://bundangestelltertieraerzte.de/jetzt-mitglied-im-bat-werden/
