# Supplementary material for: Survey on the Working Conditions, Salary, and Job Satisfaction of Employed Veterinarians in Germany
Source: Vet Sci. 2026 May 19;13(5):494. doi: 10.3390/vetsci13050494 (PMC13211543; doi:10.3390/vetsci13050494)
Supplement: Supplementary file 1 [file vetsci-13-00494-s001.zip › S4_comparison of salary to 2020.pdf]

Years within the profession	Salary of the 2025 survey (median; n=984)*	Salary of the 2020 survey (median; n=1,335)*	increase (%)
1	21.39 €	16.67 €	28.31
2	23.18 €	18.46 €	25.57
3	23.22 €	20.46 €	13.49
4	26.42 €	20.46 €	29.13
5	27.69 €	24.62 €	12.47
6	28.61 €	24.62 €	16.21
7	29.42 €	24.62 €	19.50
8-9	29.76 €	25.38 €	17.26
10-11	30.29 €	25.24 €	20.01
12-13	30.65 €	26.96 €	13.69
14-15	31.73 €	26.96 €	17.69
16-19	31.82 €	27.44 €	15.96
≥ 20	34.62 €	30.00 €	15.40
		Mean	18.82
